# Public Schools

**Published:** 1876-04

**Authors:** 


### PUBLIC SCHOOLS.

FOR rapidity of improvement and thoroughness of instruction, the public schools of the city of New-York are believed to be without equals any where. The children are educated to a promptness of speech, and thought, and action; to a system of habit and propriety of deportment which is simply wonderful to those who, for years and years, have submitted to the extravagant charges, the degrading shams, the skinnings and the skimmings of nine tenths of the private schools, academies, and institutes with which our city abounds. The pretentious character of these latter establishments is so generally conceded, that they are patronized by two classes mainly — both, however, rich; those lately so, hoping to edge their daughters into "good society," and those who are "too busy" to give their attention to the best interests of their children, or have not the intelligence necessary to determine what those best interests are; while the true "society," the wealthy and reflecting, are compelled to adopt the system of private teachers.

Those who wish to give their daughters a thorough education, have the alternative of the public school or the "governess." Unfortunately, the latter involves an expense far beyond the masses, while as to the former, thoughtful and observant men will scarcely hesitate to say that, as far as physical benefits are concerned, as far as pertains to the future well-being and happiness of the girls, to say nothing of the best interests of our community, every public school building in the city of New York had better be razed to its foundation-stone and "lightered" into the Atlantic; every teacher supplied with a sewing machine or thrifty husband, and every school commissioner and superintendent of public instruction sent to picking oakum or cracking rocks for the Central Park.

A more systematic series of machinations for the slaughter of our daughters under the guise of philanthropic efforts could not well be devised than that which prevails in the conducting of the public schools for girls in this city. These charges are sweeping, but they are literally and critically but too true.

Mother, look at your joyous and perpetual-motion daughter of four, five, or six years old. Put her in a chair, and require her to sit still, or try to keep her in the room for half an hour; nay, try it yourself, and you will find the former, at least to you, an impossible thing, and the latter intolerable. But that same child is confined to the walls of a public school from nine in the morning until three o'clock in the afternoon, and for four or five hours of that time sits on a hard bench; and for a considerable portion of these they are required to sit still under penalty that if they move foot, or hand, or head, they shall be "kept in" after three o'clock, with the disgrace of the thing patent to every eye of hundreds of their schoolmates. It is truly pitiful to look at the countenances of the little creatures as they come out from the place of stocks and thumb-screws at three o'clock of a spring or summer afternoon. There is an expression of fatigue and sad exhaustion in most of them which almost extorts a curse upon all who aid and abet the murderous inhumanity.

But these enormities become more inexcusable. Talk about the lash of a negro overseer! Parents may rest assured that they had a million times better give a tithe of their attention towards "ameliorating the condition" of their own children—a tithe of their money towards constructing an "underground railroad" for emancipating their own offspring from the infliction of moral lashings which not only kill the body, but murder the intellect and prostrate the higher nature in the dust.

These children, not having eaten any thing since about eight o'clock except a "lunch" of candy, pound-cake, gingerbread, or sandwich, which is always required to be eaten in a few minutes, and not unseldom while standing in a line, it may be readily supposed that they are very hungry by the time they get home, and are ready to sit down to dinner about four o'clock, when, almost famished and exceedingly weary, they very naturally swallow with rapidity and eat a great deal. But before the four o'clock dinner is scarce half-digested, the tea-bell of six and a half or seven rings, and they sit down again to a fill of preserves, sweet-cakes, tea-biscuit, and other delicacies, so that from four until bed-time they are more like gorged anacondas than any thing else. But what then? Do they go out to joyous play? Not a bit of it. They have "lessons to get," to which task the parents' command is not necessary to drive them. Either the fear of their teachers, or dread of disgrace in the presence of their companions, or a consuming ambition, goads them to their books, from which, if they are conscientious, they do not feel at liberty to rise, on an average, until eight, nine, or even ten o'clock, only to hurry up in the morning to tramp the same tread-mill until breakfast, all anxious to get to school in time for fear of a "mark" against them.

There is nothing like plain facts for illustration. In a house in this street, on the second day of April, in the year of progress, eighteen hundred and sixty, a girl of ten years was bending over a "geography lesson." Something was evidently the matter; the countenance was sad, dispirited, and by flashes, angry. On asking the cause, "Such a hard lesson!" On looking at it, it was found to consist of three pages of double columns, each line containing one question, and sometimes four! Questions embracing the name of the capital of each State, its situation, and a variety of other particulars, which gave the sum total of questions to be hunted out by a child of ten years of age, from four o'clock until eight next morning, of one hundred and fifty-two.

Besides this, there were two other lessons to learn, one of spelling, the other in arithmetic. The next day the lesson was not "said," because there was not time for its recitation. That is to say, there is more to be learned by the children at home, from four in the afternoon until school-time next day, than can be listened to by the teacher in the six hours from nine to three. On another occasion the lesson was so clearly beyond the ability of the children to compass, that the class, nearly a hundred, recited it so imperfectly, that half of the same lesson was given out for the next day. Such a lack of judgment on the part of teachers merits most severe condemnation.

It requires no argument to prove to any man, not an idiot, that any child must, under such a routine, wilt and wither like a flower without water. Under the circumstances, a modification of the public school system is imperative, and we call upon the two great moral leaders of the age, the pulpit and the press, to the immediate advocacy of the abolishment of home study; that no lessons, under any circumstances, be required to be learned out of school hours; and no greater evidence of progress, of advancement in intelligence and a sound policy has lately been given to the country, than in the recent action of the school commissioners of Boston, in forbidding the assignment of lessons, for study out of school, to girls—the city physician having become convinced of the alarming evils resulting from such studies. Can New-York, with all its wealth of gold, and mind, and money, and magnificent enterprise, furnish no intellect bright enough to see and expose the intolerable stupidity of our public school management? Out of the pages of this monthly and those of Fowler & Wells, to wit, *Life Illustrated*, *The Water Cure*, and *The American Phrenological Journal*, we read no word of remonstrance, of entreaty, or alarm against the startling evil!

Teachers are commended for bringing the children on so rapidly. Parents are flattered at the progress of their little ones, and school committees and superintendents are charmed with the tokens of solid advancement made by the youthful martyrs; but they take no note of the sad face, of the dray-horse look, of the heavy tread, the flushed cheek, the preternaturally bright eye, the cold fingers, and the clammy feet! Out upon such short-sighted intellects, such leaden dolts! If it is a question of education and disease, or of ignorance and glorious health for our daughters, we ourselves clutch at the union of health and ignorance with the greediness a famished tiger pounces on a fresh fat lamb. Ignorance with health may be useful, may be happy; but a finished education with a fell disease eating out the life, can be neither, and must early go down to the grave a blighted bud, a priceless jewel shivered in the polishing. But health and high development need never be dissevered. Extend the time of girlhood, of "going into society," of "husbanding," from sixteen to twenty-six. Let one study be pursued at a time; one solid study and one ornamental accomplishment, and when one is thoroughly mastered, take up a second and a third, giving, from the age of ten, three hours a day to domestic activities and superintendencies and out-door exercises; then, at twenty-five, a young man may marry a woman, not a lady—may mary a help-meet, not a puny, whining, simpering, skinny bag of bones; may marry a counselor, a coöperator, and an adviser, not a thriftless, lounging, dressy, helpless doll. The incoöperative, useless, senseless, sickly wife drives not a few men, capable of higher things, to discouragement, to the bottle, to the prison, and to the suicide's grave, because their wives were first ruined mentally and morally by the shams of dress and show learned but too facilely at the detestable "boarding-school for young ladies," or the hot houses of mental culture, the "public school." The mother molds the man; she molds the destinies of her country; but an invalid at twenty, as nine out of ten are, they can not do otherwise than bring to their country's altar, not the lambs "without blemish and without spot," but sons and daughters in body diseased, feeble in intellect, in heart and soul a shell and a sham!

